# Catalytically inactive RIP1 and RIP3 deficiency protect against acute ischemic stroke by inhibiting necroptosis and neuroinflammation

**DOI:** 10.1038/s41419-020-02770-w

**Published:** 2020-07-23

**Authors:** Yue Zhang, Ming Li, Xiaoming Li, Haiwei Zhang, Lingxia Wang, Xiaoxia Wu, Haibing Zhang, Yan Luo

**Affiliations:** 1 Department of Anesthesiology, Ruijin Hospital, Shanghai Jiao Tong University School of Medicine, 200025 Shanghai, China; 2 CAS Key Laboratory of Nutrition, Metabolism and Food Safety, Shanghai Institute of Nutrition and Health, Chinese Academy of Sciences, 200031 Shanghai, China

**Keywords:** Necroptosis, Cell death and immune response, Stroke

## Abstract

Necroptosis, which is mediated by RIP1/RIP3/MLKL (receptor-interacting protein kinase 1/receptor-interacting protein kinase 3/mixed lineage kinase domain-like protein) signaling, is a critical process in the development of acute ischemic stroke. However, it is unclear precisely how necroptosis promotes the pathogenesis of acute ischemic stroke. In this experimental study in mice, we investigated how necroptosis loss-of-function mice, RIP1 kinase-dead mice, RIP3-deficiency mice, and MLKL-deficiency mice could be protected against cerebral injury after acute ischemic stroke. Insoluble RIP1, RIP3, and MLKL were all detected in the infarct area of the study mice, indicating activation of necroptosis. Two types of RIP1 kinase-dead mutant mice (*Rip1*^*K45A/K45A*^ or *Rip1*^Δ/Δ^) were used to show that catalytically-inactive RIP1 can decrease the infarct volume and improve neurological function after MCAO/R (middle cerebral artery occlusion/reperfusion). Both *Rip3*^−/−^ mice and *Mlkl*^−/−^ mice were protected against acute ischemic stroke. In addition, necroptosis loss-of-function mice showed less inflammatory responses in the infarct area. Therefore, necroptosis and its accompanying inflammatory response can lead to acute injury following ischemia stroke. Our study provides new insight into the pathogenetic mechanisms of acute ischemic stroke, and suggests potential therapeutic targets for neuroprotection.

## Introduction

Acute ischemic stroke is a common disease throughout the world with high mortality rates and high disability rates^1^. In the pathogenesis of acute ischemic stroke, multiple cell death mechanisms occur, including necrosis, apoptosis, and autophagy^2,3^. Previously, necrosis was considered a type of non-programed cell death differing from apoptosis, which is regulated by caspase cascades. However, recent studies have demonstrated that necrotic cell death can also be a regulated process, which is known as necroptosis^4–7^. Necroptosis is mediated by necrosome-containing receptor-interacting protein kinase 1 (RIP1), receptor-interacting protein kinase 3 (RIP3), and mixed lineage kinase domain-like protein (MLKL). In certain pathological conditions, RIP1 will be activated and combine with RIP3 via a RIP homotypic interaction motif (RHIM) domain to form an amyloid complex, known as complex IIb. The activated RIP3 in complex IIb further phosphorylates the downstream molecule MLKL, which forms oligomers and aggregates on the plasma membrane to cause necroptosis through disruption of plasma membrane and cell lysis. Subsequently, a large amount of cellular contents such as damage-associated molecular patterns (DAMPs) are released, causing a secondary inflammatory reaction which aggravates the tissue damage^4,8–10^.

Previous studies have verified that markers of necroptosis are increased in the ipsilateral striatum of the rat brain in the middle cerebral artery occlusion/reperfusion (MCAO/R) model^11–13^. Pharmacological data have also demonstrated that necrostatin-1 (Nec-1), a necroptosis inhibitor, reduces infarct volumes and promotes neurological function recovery in rodents that have suffered an acute ischemic stroke^14,15^. These findings suggest that necroptosis might be involved in the pathogenesis of acute ischemic stroke. However, the precise mechanism of how necroptosis promotes the pathogenesis of acute ischemic stroke is still unclear.

Neuroinflammation is a key driver of neuron damage and neurodegeneration in acute ischemic stroke. The levels of pro-inflammatory cytokines and chemokines, including tumor necrosis factor-α (TNF-α), interleukin-1β (IL-1β), interleukin-6 (IL-6) and C-C motif chemokine ligand 2 (CCL2) are elevated after acute cerebral ischemia and reperfusion injury^16^. In addition, several inflammatory-associated cell signaling cascades such as the mitogen-activated protein kinase (MAPK) pathway and the nuclear factor-κB (NF-κB) pathway are involved in the pathogenesis of acute ischemic stroke^17,18^. Previous studies have demonstrated that RIP1 kinase activation also leads to neuroinflammation in multiple central nervous system (CNS) diseases such as Alzheimer′s disease (AD)^19^ and amyotrophic lateral sclerosis (ALS)^20^. In addition, it is widely known that RIP3 also plays an important role in inflammatory regulation^21–26^. However, the impact of RIP1 kinase activation and RIP3 on neuroinflammation in acute ischemic stroke has not been demonstrated. In this experimental study, we employed RIP1 kinase-dead mice, RIP3-deficiency mice, and MLKL-deficiency mice to evaluate the impacts of RIP1, RIP3, and MLKL in acute ischemic stroke.

## Results

### Necroptosis is activated in the infarct area after MCAO/R

**Fig. 1:**
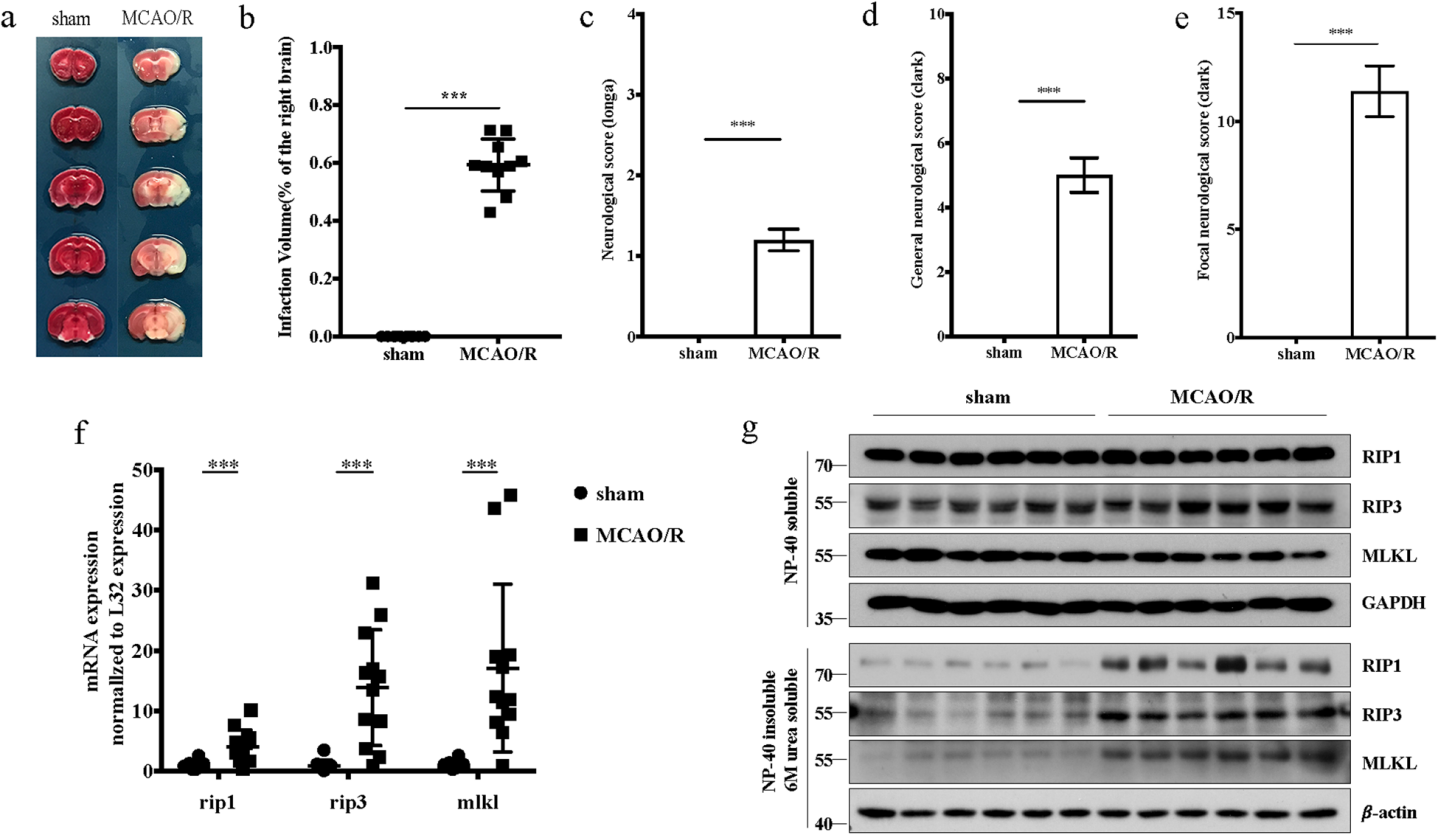
Necroptosis is activated in the infarct area after MCAO/R. **a** Representative TTC-stained images of brain slices in *WT* mice in the sham group and the MCAO/R group. **b** Comparison of the percentages of infarct volume between the sham group and the MCAO/R group. Each symbol represents one male mouse (sham, *n* = 10; MCAO/R, *n* = 10). Bars indicate the means ± SEM; the *P*-value was determined using a *t*-test; ***P < 0.001. **c**–**e** Comparison of the neurological scores between the sham group and the MCAO/R group (n = 10): Longa score (**c**), general Clark score (**d**), and focal Clark score (**e**). Data in bar graphs are means ± SEM; *t*-test: ***P < 0.001. **f** mRNA expression of RIP1, RIP3, and MLKL in the infarct were increased area at 24 h after MCAO/R. Each symbol represents one male mouse (sham, *n* = 12; MCAO/R, *n* = 12). Bars indicate the means ± SEM; *t*-test: ***P < 0.001. **g** Increased levels of insoluble RIP1, RIP3, and MLKL were found in the mouse infarct area at 24 h after MCAO/R (*n* = 6).

To investigate the pathogenesis of acute ischemic stroke, we firstly established the MCAO/R model to simulate cerebral ischemia-reperfusion injury. TTC (2,3,5-triphenyltetrazolium chloride) staining showed that the infarct volume increased significantly at 24 h after MCAO/R (Fig. 1a, b). In addition, two types of neurobehavioral scoring systems (those reported by Longa et al.^27^ and Clark et al.^28^) were used to evaluation mouse neurological function. Both scoring systems indicated that neurological function was impaired after MCAO/R (Fig. 1c–e).

We then examined the necroptosis-related gene (*RIP1, RIP3*, and *MLKL*) expression in the infarct area after MCAO/R. RT-PCR results showed that mRNA expression levels of RIP1, RIP3 and MLKL were significantly increased in the ipsilateral hemisphere (Fig. 1f). Previous studies have shown that RIP1 and RIP3 form insoluble amyloid-like structures when necroptosis is activated^29^. Necroptosis can be assessed by examining the differential solubilities of RIP1, RIP3 and MLKL within tissues^20,30,31^. We therefore lysed proteins using buffer with NP-40 and 6M-urea successively, and found that RIP1, RIP3 and MLKL levels in the 6M-urea fraction were elevated in the MCAO/R group in comparison with the sham group of mice (Fig. 1g). These results are similar to earlier studies showing necroptosis activation in other CNS diseases such as ALS, MS (multiple sclerosis), and AD^20,30,31^. Collectively, these results suggest that necroptosis is activated in the infarct area after MCAO/R.

### RIP1 kinase-dead mutants have decreased infarct volumes and improved neurological function after MCAO/R

**Fig. 2:**
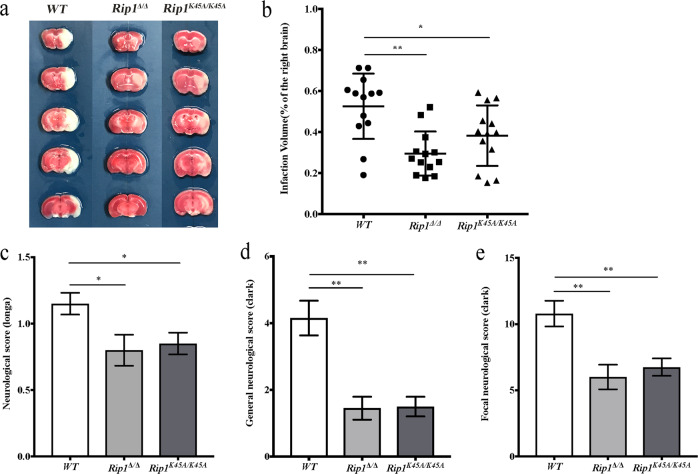
RIP1 kinase-dead mutants have decreased infarct volumes and improved neurological function after MCAO/R. **a** Representative TTC-stained images of brain slices in *WT*, *Rip1*^Δ/Δ^, and *Rip1*^*K45A/K45A*^ mice at 24 h after MCAO/R. **b** Comparison of the percentages of infarct volume between *WT* mice and *Rip1*^Δ/Δ^*/Rip1*^*K45A/K45A*^ mice. Each symbol represents one male mouse (*n* = 13). Bars indicate the means ± SEM; *P*-values were determined using a *t*-test: *P < 0.05; **P < 0.01. **c**–**e** Comparison of neurological scores between *WT* mice and *Rip1*^Δ/Δ^*/Rip1*^*K45A/K45A*^ mice at 24 h after MCAO/R (*n* = 20): Longa score (**c**), general Clark score (**d**), and focal Clark score. **e** Data in bar graphs are means ± SEM; *t*-test: *P < 0.05; **P < 0.01.

To investigate the role of RIP1 kinase activity in acute ischemic stroke, two types of RIP1 kinase-dead mice (*Rip1*^*K45A/K45A*^ and *Rip1*^Δ/Δ^ mice^32^) were investigated by the MCAO/R model. In comparison with *WT* (wild-type) mice, infarct volumes in both *Rip1*^*K45A/K45A*^ mice and *Rip1*^Δ/Δ^ mice were significantly reduced (Fig. 2a,b). Consistent with this, RIP1 kinase activity deficiency significantly attenuated both the Longa and Clark scores (Fig. 2c–e), suggesting that RIP1 kinase activity deficiency improved the neurological function of mice after MCAO/R injury. Together, these results indicate that RIP1 kinase-dead mice are protected against acute ischemic stroke.

### RIP1 kinase-dead mutants have decreased cell death and inflammatory responses in the infarct area

**Fig. 3:**
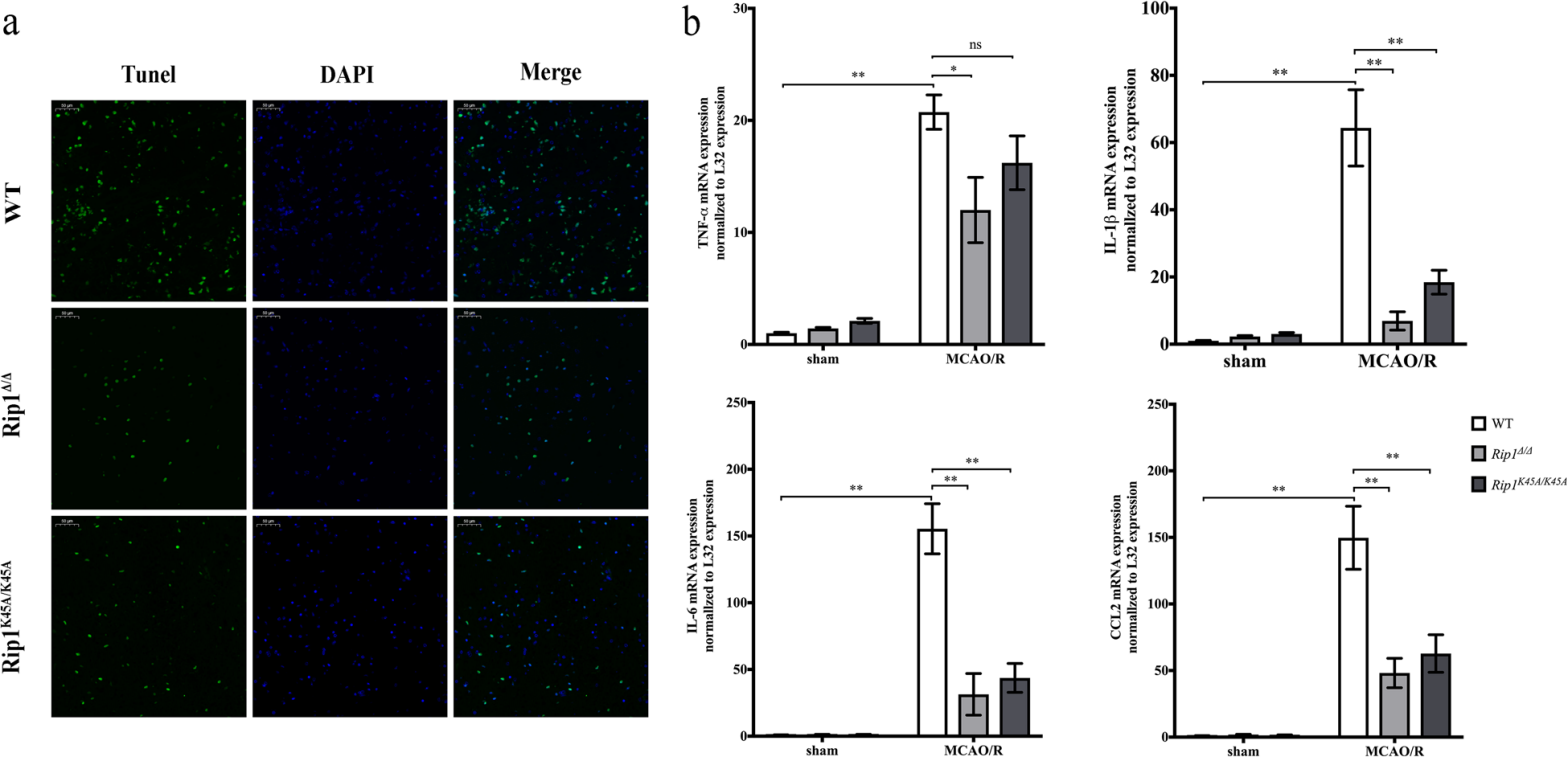
RIP1 kinase-dead mutants show decreased cell death and inflammatory responses in the infarct area. **a** TUNEL staining of brains in *WT*, *Rip1*^Δ/Δ^, and *Rip1*^*K45A/K45A*^ mice at 24 h after MCAO/R. TUNEL (green) was used for death cell staining and DAPI (blue) for nuclear staining. Scale bar, 50 μm. **b** mRNA expression of cytokines and chemokines in infarct brain tissues from *WT*, *Rip1*^Δ/Δ^, and *Rip1*^*K45A/K45A*^ mice in the sham group and the MCAO/R group (*n* = 5–6). Data in bar graphs are means ± SEM; *t*-test: ns, not significant; *P < 0.05; **P < 0.01.

We next examined cell death in the infarct area by TUNEL (terminal deoxynucleotidyl transferase dUTP nick end labeling) staining. The results showed that both *Rip1*^*K45A/K45A*^ mice and *Rip1*^Δ/Δ^ mice had decreased cell death in the infarct area (Fig. 3a). Several previous studies have confirmed that neuroinflammation is a critical process in acute ischemic stroke^16–18^. In this study, we used RT-PCR to detect cytokine and chemokine expression in the infarct area and found that this was significantly increased in the MCAO/R group (Fig. 3b). However, serum levels of these inflammatory mediators detected by ELISA showed no significant differences between the sham group and the MCAO/R group (Supplementary Fig. S1). These results suggest that the timepoint of 1 h for MCAO and 24 h for reperfusion only causes inflammatory responses in focal brain tissues rather than a systemic inflammatory response. Therefore, we next examined the expression of TNF-α, IL-1β, IL-6, and CCL2 in the infarct areas of *WT* and RIP1 kinase-dead mice. In comparison with *WT* mice, the expression of TNF-α, IL-1β, IL-6 and CCL2 were decreased significantly in *Rip1*^Δ/Δ^ mice (Fig. 3b). Similarly, the expression of IL-1β, IL-6 and CCL2, though not that of TNF-α, were also decreased in *Rip1*^*K45A/K45A*^ mice (Fig. 3b). These results indicate that RIP1 kinase-dead mutant mice have decreased neuroinflammation in the infarct area after MCAO/R.

### RIP1 kinase-dead mutants show decreased necroptosis and activation of the NF-κB signal pathway in the infarct area

**Fig. 4:**
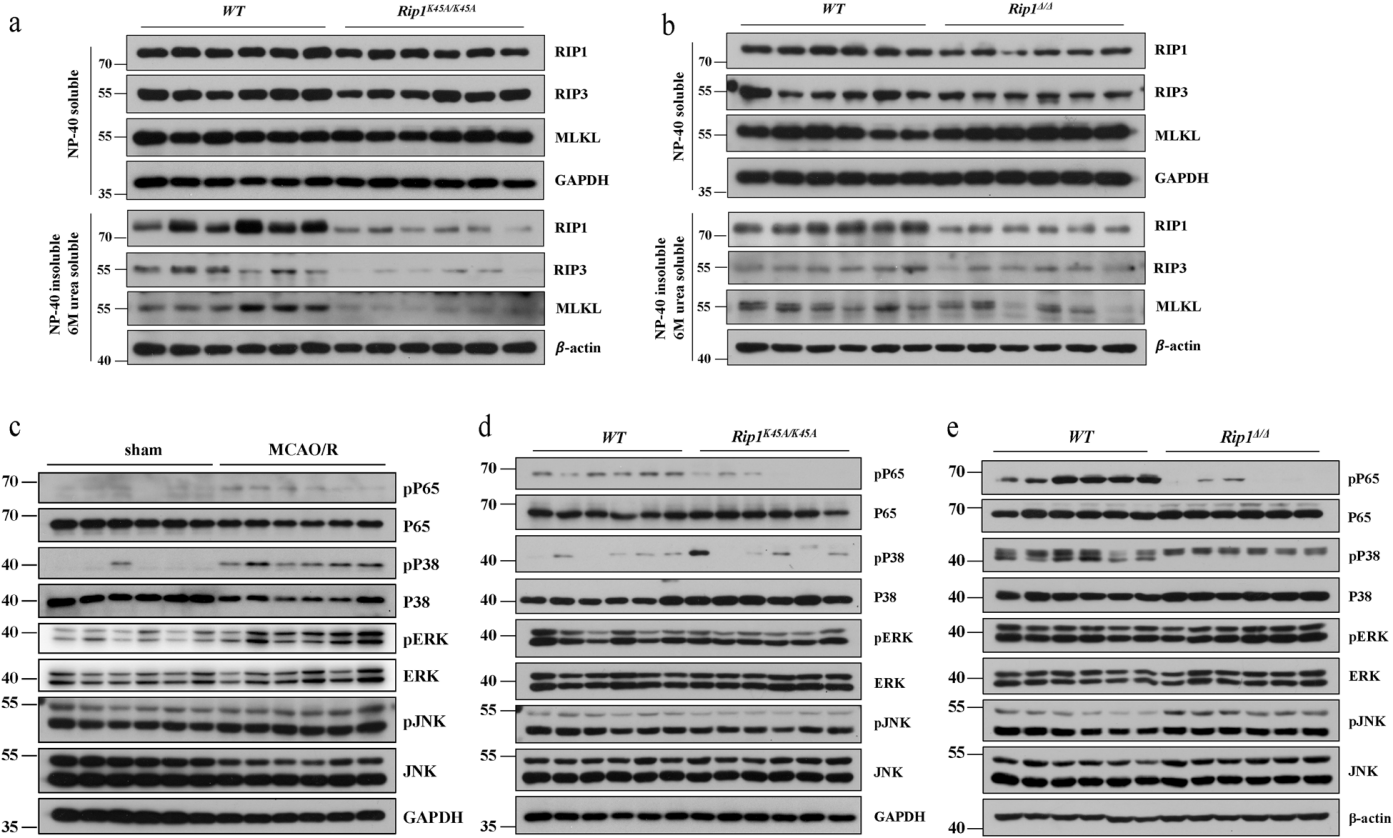
RIP1 kinase-dead mutants show decreased necroptosis and activation of the NF-κB signal pathway in the infarct area. **a** Insoluble RIP1, RIP3 and MLKL levels were decreased in the infarct area of *Rip1*^*K45A/K45A*^ mice (*n* = 6) after MCAO/R compared with *WT* mice (*n* = 6). **b** Insoluble RIP1, RIP3, and MLKL levels were decreased in the infarct area of *Rip1*^Δ/Δ^ mice (*n* = 6) after MCAO/R compared with *WT* mice (*n* = 6). **c** Activation of the NK-κB (P65) signal pathway and MAPK (P38, ERK, JNK) signal pathway in the infarct area of *WT* mice in the sham group and the MCAO/R group (*n* = 6). **d**, **e** Comparison of NK-κB and MAPK pathway activation in the infarct area of *WT* mice and *Rip1*^Δ/Δ^*/Rip1*^*K45A/K45A*^ mice (*n* = 6).

To further study the protective mechanisms in RIP1 kinase-dead mutant mice in acute ischemic stroke, levels of RIP1, RIP3, and MLKL were detected in the infarct areas of *WT* mice and RIP1 kinase-dead mutants (*Rip1*^*K45A/K45A*^ mice and *Rip1*^Δ/Δ^ mice). As shown in Fig. 4a, b, *Rip1*^*K45A/K45A*^ mice and *Rip1*^Δ/Δ^ mice had decreased levels of RIP1, RIP3, and MLKL in the insoluble 6 M urea fraction, suggesting that in RIP1 kinase-dead mutants the necroptosis process in the infarct area is prevented after MCAO/R. Moreover, the activity of the NF-κB signal pathway and the MAPK signal pathway were examined by immunoblotting. We found that P65, P38 and ERK (extracellular signal-regulated kinase) were activated after MCAO/R, but the phosphorylation of JNK (c-Jun N-terminal kinase) showed no difference between the sham group and the MCAO/R group (Fig. 4c). We next tested if the inflammatory signaling pathways are disrupted in *Rip1*^*K45A/K45A*^ mice or *Rip1*^Δ/Δ^ mice after acute ischemic stroke. Our results showed that both *Rip1*^*K45A/K45A*^ mice and *Rip1*^Δ/Δ^ mice have decreased the phosphorylation of P65 without affecting other signal pathways (Fig. 4d, e). Collectively, these results indicate that RIP1 kinase-dead mutant mice exhibit decreased necroptosis and activation of the NF-κB signal pathway in the infarct area.

### RIP3 deficiency protects against cerebral damage after MCAO/R by attenuating necroptosis and neuroinflammation

**Fig. 5:**
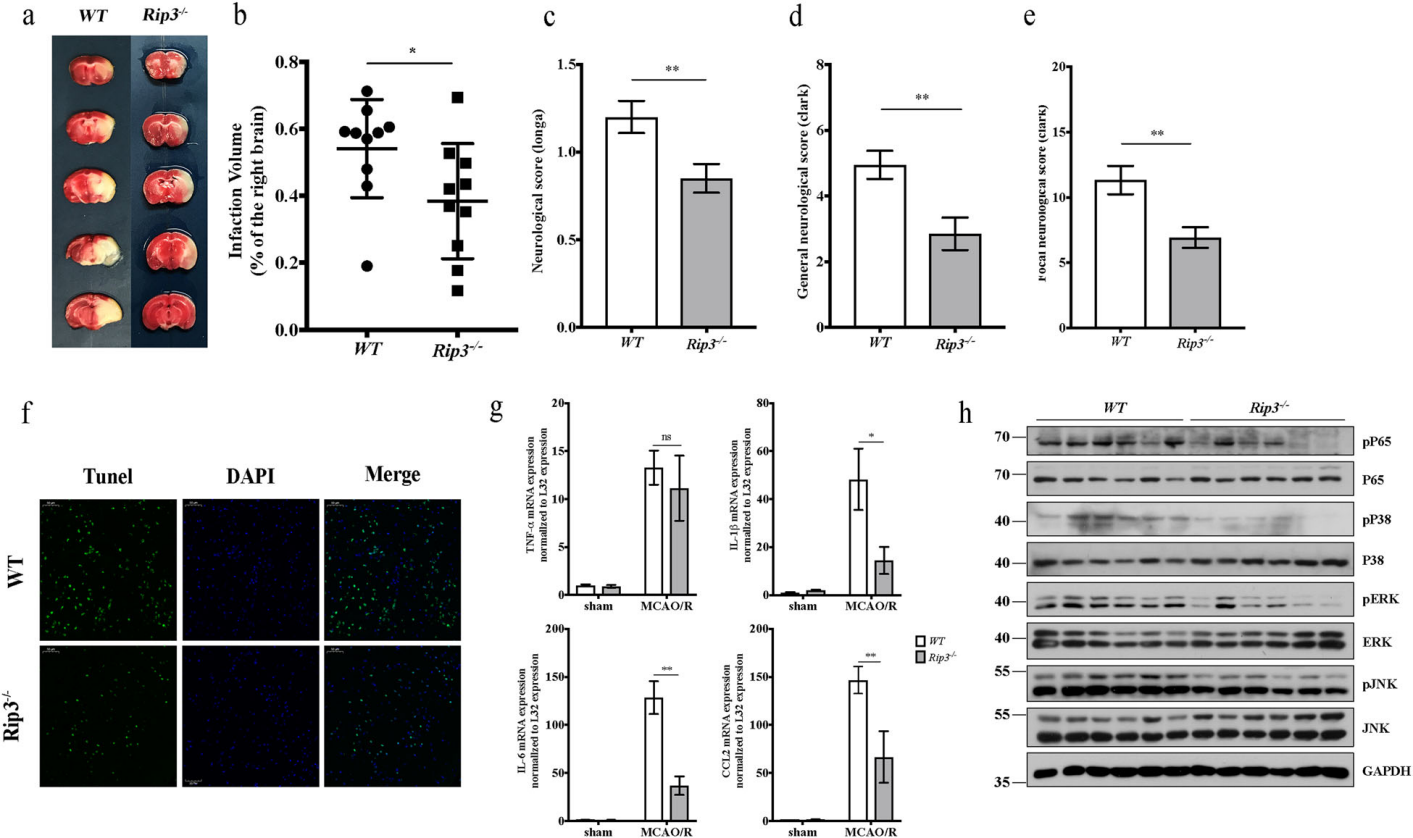
RIP3 deficiency protects against cerebral damage after MCAO/R by attenuating necroptosis and neuroinflammation. **a** Representative TTC-stained images of brain slices in *WT* mice and *Rip3*^−/−^ mice at 24 h after MCAO/R. **b** Comparison of the percentages of infarct volume between *WT* mice and *Rip3*^−/−^ mice. Each symbol represents one male mouse (*n* = 10). Bars indicate the means ± SEM; *P*-values were determined using a *t*-test: *P < 0.05**. c–e** Comparison of neurological scores between *WT* mice and *Rip3*^−/−^ mice at 24 h after MCAO/R (*n* = 20): Longa score (**c**), Clark general score (**d**), and Clark focal score (**e**). Data in bar graphs are means ± SEM; *t*-test: ns not significant; **P < 0.01. **f** TUNEL staining of the infarct area of *WT* and *Rip3*^−/−^ mice after MCAO/R. Scale bar, 50 μm. **g** Cytokine and chemokine expressions in the infarct area from *WT* mice and *Rip3*^−/−^ mice in the sham group and the MCAO/R group were detected by RT-PCR (*n* = 6). Data in bar graphs are means ± SEM; *t*-test: ns not significant; *P < 0.05, **P < 0.01. **h** Brain samples of the infarct area from *WT* mice and *Rip3*^−/−^ mice were analyzed by immunoblotting to examine activation of the NK-κB pathway and MAPK pathway (*n* = 6).

To further explore the role of RIP3 in acute ischemic stroke, RIP3-deficiency mice and *WT* mice were subjected to MCAO/R. The infarct volumes and neurological scores were detected at 24 h after MCAO/R. In comparison with *WT* mice, the infarct volume in *Rip3*^−/−^ mice was significantly reduced (Fig. 5a, b). Consistent with this, the neurological score was significantly decreased in *Rip3*^−/−^ mice, indicating that RIP3 deficiency attenuated neurological function damage after MCAO/R (Fig. 5c–e).

Previous studies have shown that RIP3 is not only an essential mediator of necroptosis, but also a regulator of inflammatory responses^21–26^. Therefore, we next examined cell death by TUNEL staining. The result showed that *Rip3*^−/−^ mice exhibited decreased cell death in the infarct area (Fig. 5f). In comparison with *WT* mice, *Rip3*^−/−^ mice also showed decreased expression of IL-1β, IL-6, and CCL2 mRNA in the infarct area, though not expression of TNF-α (Fig. 5g). In addition, RIP3 deficiency mice showed less phosphorylation of P65, P38 and ERK in the infarct area (Fig. 5h). Collectively, these results indicate that RIP3 deficiency protects against cerebral damage after MCAO/R by attenuating necroptosis and neuroinflammation.

### MLKL deficiency alleviates cerebral injury after MCAO/R by preventing necroptosis without affecting inflammatory-related signal pathways

**Fig. 6:**
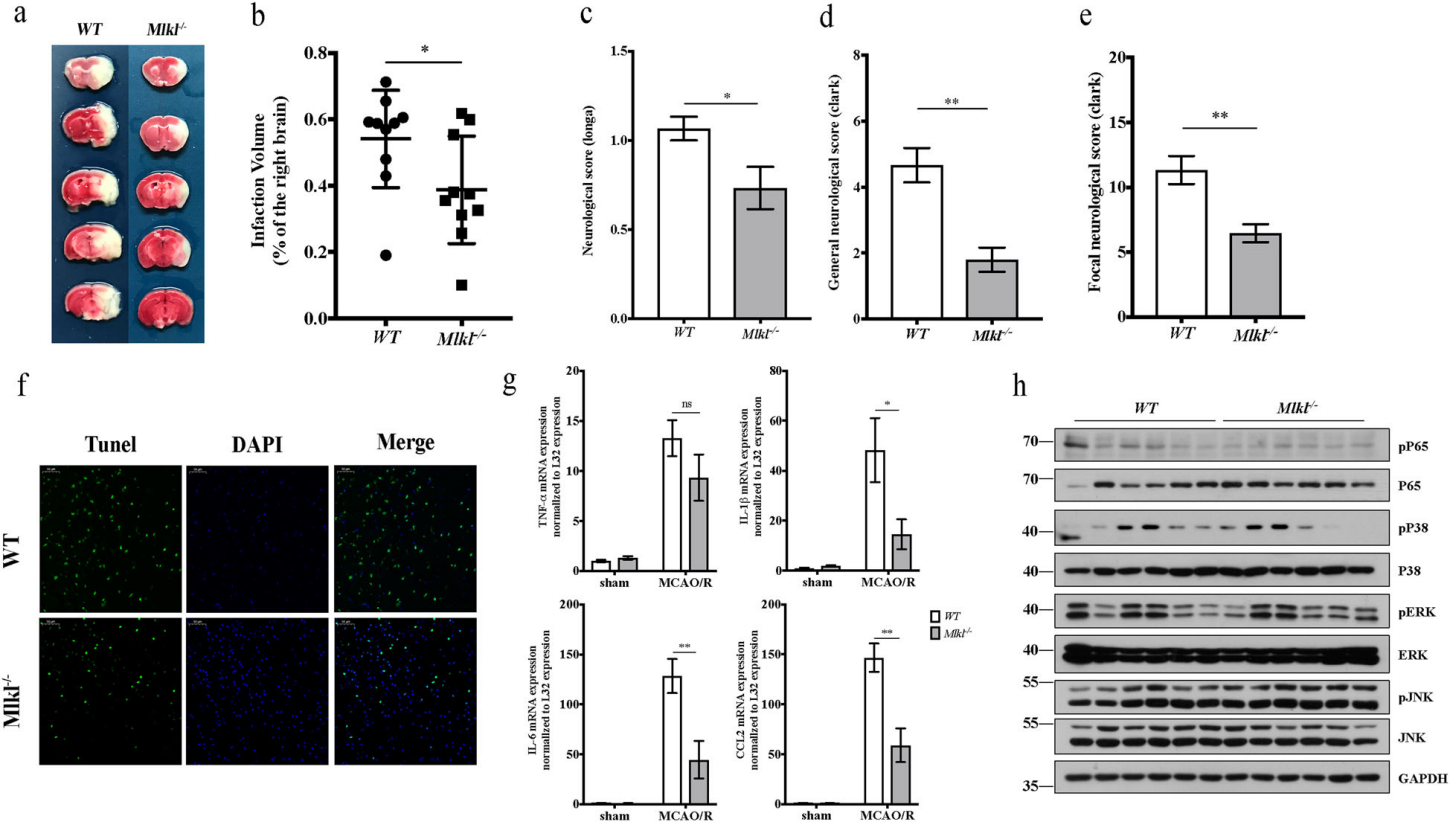
MLKL deficiency attenuates cerebral injury after MCAO/R by preventing necroptosis without affecting inflammatory-related signal pathways. **a** Representative TTC-stained images of brain slices in *WT* mice and *Mlkl*^−/−^ mice after MCAO/R. **b** Comparison of percentages of infarct volumes between *WT* mice and *Mlkl*^−/−^ mice. Each symbol represents one male mouse (*n* = 10). Bars indicate the means ± SEM; *P*-values were determined using a *t*-test: *P < 0.05. **c**–**e** Neurological function was compared via neurological scores between *WT* mice and *Mlkl*^−/−^ mice after MCAO/R (*n* = 15): Longa score (**c**), general Clark score (**d**), and focal Clark score (**e**). Data in bar graphs are means ± SEM; *t*-test: *P < 0.05; **P < 0.01. **f** TUNEL staining of the infarct area in *WT* and *Mlkl*^−/−^ mice after MCAO/R. Scale bar,50 μm. **g** mRNA expression of cytokines and chemokines in the infarct area from *WT* mice and *Mlkl*^−/−^ mice in the sham group and the MCAO/R group (*n* = 5–6). Data in bar graphs are means ± SEM; *t*-test: ns not significant; *P < 0.05; **P < 0.01. **h** Activation of the NK-κB pathway and MAPK pathway in infarct areas from *WT* mice and *Mlkl*^−/−^ mice were examined by immunoblotting (*n* = 6).

MLKL is the critical terminal executioner of necroptosis. Therefore, we used MLKL-deficient mice to determine the effect of MLKL in acute ischemic stroke. TTC staining showed that the infarct volume in *Mlkl*^−/−^ mice was significantly lesser than that in *WT* mice (Fig. 6a, b). Consistent with this, *Mlkl*^−/−^ mice also showed improved neurological function after MCAO/R (Fig. 6c–e). These data indicate that MLKL deficiency alleviates cerebral injury after MCAO/R. Furthermore, *Mlkl*^−/−^ mice also exhibited decreased cell death and release of cytokines and chemokines in the infarct area (Fig. 6f, g). However, there appeared to be no effect on NF-κB and MAPK signal pathways in *Mlkl*^−/−^ mice after acute ischemic stroke, as assessed by the phosphorylation of P65, JNK, ERK and P38 in the infarct area (Fig. 6h). Taken together, these data suggest that MLKL deficiency alleviates cerebral injury after MCAO/R by inhibiting necroptosis without affecting inflammatory-related signal pathways. We speculate that the reduction of inflammatory factors in *Mlkl*^−/−^ mice after MCAO/R might be caused by decreased release of cellular contents after necroptosis.

## Discussion

Once necroptosis is induced, activated RIP1 and RIP3 form amyloid-like fibrils^29^. Insoluble activated RIP1, RIP3, and MLKL can be found in multiple CNS diseases in which necroptosis is involved^20,30,31^. In prior studies, phosphorylation of necroptosis makers has been used to indicate activation of necroptosis^11,12,33^. In our study, we found firstly that insoluble RIP1, RIP3, and MLKL were increased in the infarct area of mice suffering from MCAO/R injury, convincingly showing that necroptosis is activated in acute ischemic stroke.

Previous studies have reported that necrostatin-1 protects against cerebral injury after acute ischemic stroke by preventing RIP1/RIP3/MLKL-mediated necroptosis^11,14,15^. Consistent with these studies, we firstly employed two types of RIP1 kinase-dead mice (*Rip1*^*K45A/K45A*^ mice and *Rip1*^Δ/Δ^ mice) to demonstrate that inhibiting RIP1 kinase activity decreased the infarct area and improved neurological function after MCAO/R. Similar to RIP1 kinase-dead mice, we also showed that *Rip3*^−/−^ mice and *Mlkl*^−/−^ mice have diminished cerebral damage after MCAO/R. These results further demonstrate that necroptosis plays an essential role in the pathogenesis of acute ischemic stroke, and suggest that inhibiting the necroptosis process could significantly alleviate cerebral injury after MCAO/R. Our results are similar to that those of a recent study^33^ that used photothrombosis to induce permanent cerebral ischemic injury. However, Newton et al.^34^ reported that *RIP3*^−/−^ mice didn′t show attenuated brain injury following MCAO^34^. We speculate that different experimental conditions or backgrounds between particular animal facilities may account for these differing results.

Previous study reported by Liu et al. showed that *Rip1*^Δ/Δ^ mice is more effective than *Rip1*^*K45A/K45A*^ mice in restoring embryonic lethality caused by FADD deficiency, which might attribute to their extents of inhibition on necroptosis^32^. In our study, we found that both RIP1 kinase-dead mutant mice(*Rip1*^*K45A/K45A*^ or *Rip1*^Δ/Δ^) had protective effects against cerebral injury after ischemic stroke, which were reflected in the reduction of infarct area and neurobehavioral score. However, from the available data, there was a trend that the infarct area of *Rip1*^Δ/Δ^ mice was less than that in *Rip1*^*K45A/K45A*^ mice, although there is no significant statistical difference between them. This trend might attribute to that *Rip1*^Δ/Δ^ mice are more resistant to necroptosis than *Rip1*^*K45A/K45A*^ mice. Nevertheless, there was no significant difference between these two kinds of mice in the behavioral scores by reason that the behavioral score was the result of a combination of multiple factors.

In addition to mediating necroptosis, RIP1 kinase activity is also essential for neuroinflammation in CNS diseases^35^. Consistent with these studies, we found that RIP1 kinase-dead mice had decreased activation of the NF-κB signal pathway. In contrast to RIP1 kinase-dead mice, RIP3 deficiency affected the activation of both the NF-κB and MAPK signal pathways. These results demonstrate that RIP1 and RIP3 mediate neuroinflammation in acute ischemic stroke through different mechanisms. These mechanisms need to be investigated further. Unlike RIP1 kinase-dead mice and RIP3-deficiency mice, MLKL deficiency had no effect on either the MAPK pathway or the NF-κB pathway in acute ischemic stroke. Nevertheless, we observed that inflammatory factors were decreased in the infarct area of *Mlkl*^−/−^ mice. These results taken together suggest that MLKL deficiency might decrease inflammatory factors indirectly via inhibiting necroptosis without impacting inflammation-related signal pathways.

After MCAO/R, the mRNA expression of TNF-α in the infarct area showed no significant difference between *WT* group and *Rip1*^*K45A/K45A*^ group, as well as *Rip3*^−/−^ and *Mlkl*^−/−^ group. We next examined the inflammatory factors at protein level by ELISA and found that necroptosis loss-of-function mice(*Rip1*^*K45A/K45A*^*, Rip1*^Δ/Δ^*, Rip3*^−/−^ and *Mlkl*^−/−^ mice) decreased TNF-α, IL-1β and IL-6 expression at protein level (Supplementary Fig. S2). Consistent with the results at the mRNA level, these data further suggest that necroptosis loss function mice could reduce inflammatory responses in infarct area of mice after ischemic stroke. The difference results between protein levels and mRNA levels might due to time points.

Although this study showed that necroptosis loss function mice reduce infarct damage and display less inflammatory responses, it could not prove the causal relationship of them. It is well known that when cells die, they will release numerous cell contents, such as DAMPs, which will stimulate other cells, including macrophages, to further secrete inflammatory factors leading to an inflammatory response. At the same time, excessive release of inflammatory factors can also lead to cell death and tissue damage. We thought that experiments using mice with cell-specific deletion of the necroptosis genes will be needed to address this issue in the future.

In conclusion, our findings indicate that necroptosis has a critical role in acute ischemic stroke. RIP1 kinase-dead mutants, RIP3 deficiency, and MLKL deficiency protect against acute ischemic stroke by blocking necroptosis. In addition, RIP1 kinase activity and RIP3 also decrease the activation of inflammatory-related signal pathways in acute ischemic stroke. Thus, necroptosis has the potential to become a therapeutic target for neuroprotection in victims of acute ischemic stroke.

## Materials and methods

### Reagents

The following antibodies were used for western blotting: RIP1 (BD Biosciences, Franklin Lakes, NJ, USA), RIP3 (ProSci, San Diego, CA, USA), MLKL (Abcam, Cambridge, UK), p-ERK (Cell Signaling Technology, Danvers, MA, USA),ERK (Cell Signaling Technology, Danvers, MA, USA), p-P38 (Cell Signaling Technology, Danvers, MA, USA), P38 (Cell Signaling Technology, Danvers, MA, USA), p-P65 (Cell Signaling Technology, Danvers, MA, USA), p-JNK (Cell Signaling Technology, Danvers, MA, USA), JNK (Cell Signaling Technology, Danvers, MA, USA),β-actin, and GAPDH (Sigma).

### Mice

Mice were housed in a specific pathogen-free facility. *Rip1*^Δ/Δ^ mice, *Rip1*^*K45A/K45A*^ mice, *Rip3*^*−/−*^ mice, and *Mlkl*^−/−^ mice were provided by Dr Haibing Zhang (SIBS, Shanghai, China). Animals were subsequently backcrossed on a C57BL/6 background for at least 8 generations. Animal experiments were conducted in accordance with the guidelines of the Institutional Animal Care and Use Committee of the Institute of Nutrition and Health, Shanghai Institutes for Biological Sciences, Chinese Academy of Sciences (CAS), and University of Chinese Academy of Sciences.

### The middle cerebral artery occlusion/reperfusion model

Focal cerebral ischemia and reperfusion injury was induced by MCAO/R in mice using an intraluminal filament technique, as described previously^36^. In brief, adult male mice weighing 22.5–24 g were fasted for 12 h but were allowed free access to water before surgery. Anesthesia was induced with 4% isoflurane and was maintained with 2% isoflurane delivered by a mask. The right middle cerebral artery of the mouse was occluded by the use of a coagulated external carotid artery stump with a nylon filament suture coated by silicone at the head end. After 60 min of occlusion, the filament was withdrawn to allow blood reperfusion. Sham-operated mice underwent the identical surgery apart from insertion of the suture to the artery. Body temperature was maintained at 37.0 ± 0.5 °C with a heating pad during the whole process. Neurological deficits in the mice after reperfusion were evaluated by the Longa et al.^27^ method, and mice were excluded from the study if the Longa score was 0 or 4. Mice were sacrificed at the indicated time.

### Neurobehavioral evaluation

At 24 h after reperfusion, the 6-point scoring system reported by Longa et al.^27^ (with modifications), and the 56-point scoring system reported by Clark et al.^28^ were used for neurological assessment by a blinded observer.

### TTC staining and infarct volume assessment

The procedures used were as described previously^37^. Briefly, at 24 h after MCAO/R, mice were sacrificed and the brains were cut into five 1 mm thick coronal slices. Sections were stained with TTC (Sigma) for 15 min at 37 °C, and fixed in 4% paraformaldehyde (PFA) overnight. The images were taken by a digital camera, and the infarct volumes were blindly analyzed with Photoshop software. Infarct sizes were expressed as percentages of the contralateral structures.

### RT-PCR

Total RNA was extracted using TRIzol reagent (Life Technologies), according to the manufacturer′s instructions. After quantification, 2 μg total RNA was reverse transcribed to complementary DNA (Takara). Transcript levels of indicated genes were quantified by quantitative RT-PCR on an ABI 7500 real-time PCR instrument with SYBR^®^ Green. Relative expression was calculated using LC32 as an internal control, as indicated. The sequences of primers was as follows:

mRIP1: 5′-GACAGACCTAGACAGCGGAG-3′ and 5′-CCAGTAGCTTCACCACTCGAC-3′;

mRIP3: 5′-CAGTGGGACTTCGTGTCCG-3′ and 5′-CAAGCTGTGTAGGTAGCACATC-3′;

mMLKL: 5′-TTAGGCCAGCTCATCTATGAACA-3′ and 5′-TGCACACGGTTTCCTAGACG-3′;

mIL-1β: 5′-CCCAACTGGTACATCAGCAC-3′ and 5′-TCTGCTCATTCACGAAAAGG-3′;

mTNF: 5′-CCCACTCTGACCCCTTTACT-3′ and 5′-TTTGAGTCCTTGATGGTGGT-3′;

mIL-6: 5′-CGGAGAGGAGACTTCACAGA-3′ and 5′-CCAGTTTGGTAGCATCCATC-3′;

mCCL2: 5′-TGAATGTGAAGTTGACCCGT-3′ and 5′-AAGGCATCACAGTCCGAGTC-3′.

### Western blot analysis

The lysis protocol was as described previously^38^. Briefly, brain tissues were dissected at 24 h after reperfusion as described previously. Subsequently, tissues were lysed in buffer with NP-40 and centrifuged for 20 min at 12000 g. The lysis part was then separated and the pellet was washed with lysis buffer and lysed with 6M-urea. Both parts of the protein were quantified by BCA kit (Thermo Scientific), and then mixed with SDS sample buffer and boiled at 95 °C for 5 min. The samples were separated using SDS-PAGE (sodium dodecyl sulphate-polyacrylamide gel electrophoresis), and transferred to a PVDF (polyvinylidene difluoride) membrane (Millipore) with 110 v for 1.5 h. The proteins were detected by using a chemiluminescent substrate (Thermo Scientific).

### ELISA assay

TNF-α, IL-1β, IL-6, and CCL2 expression in serum were detected using the ELISA KIT (eBioScience) according to the manufacturer′s instruction. Absorption at 450 nm was determined with a microplate reader (Bio-Rad, iMark, USA), and the concentrations of TNF-α, IL-1β, IL-6, and CCL2 were determined according to the standard curve generated at the same time.

### TUNEL assay

A TUNEL assay was used to detect dead cells with DNA fragmentation using an In situ Cell Death Detection Kit, POD (Roche) according to the manufacturer′s protocol.

### Statistical analysis

Data presented in this article are representative results of at least three independent experiments. The statistical significance of data was evaluated by Student′s *t*-test and the statistical calculations were performed with GraphPad Prism software.

## Supplementary Information

Supplementary Figure 1

Supplementary Figure 2

Supplementary Figure legends
