# Supplementary figures and images for: Catalytically inactive RIP1 and RIP3 deficiency protect against acute ischemic stroke by inhibiting necroptosis and neuroinflammation
Source: Cell Death Dis. 2020 Jul 23;11(7):565. doi: 10.1038/s41419-020-02770-w (PMC7378260; doi:10.1038/s41419-020-02770-w)

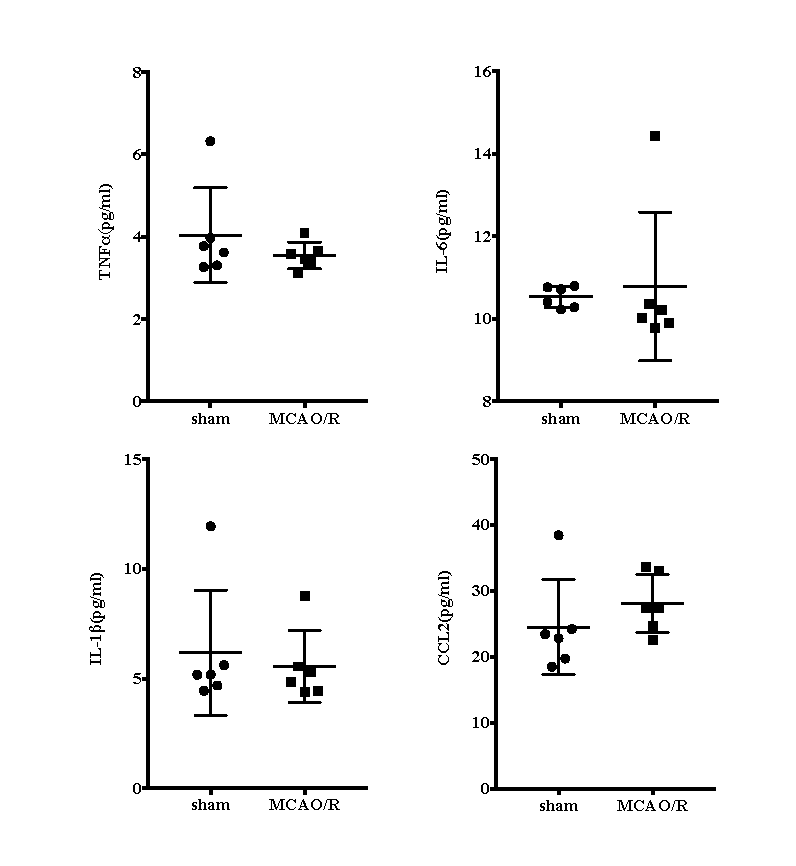

Supplement: Supplementary file 1 — Supplementary Figure 1 [file 41419_2020_2770_MOESM1_ESM.png]

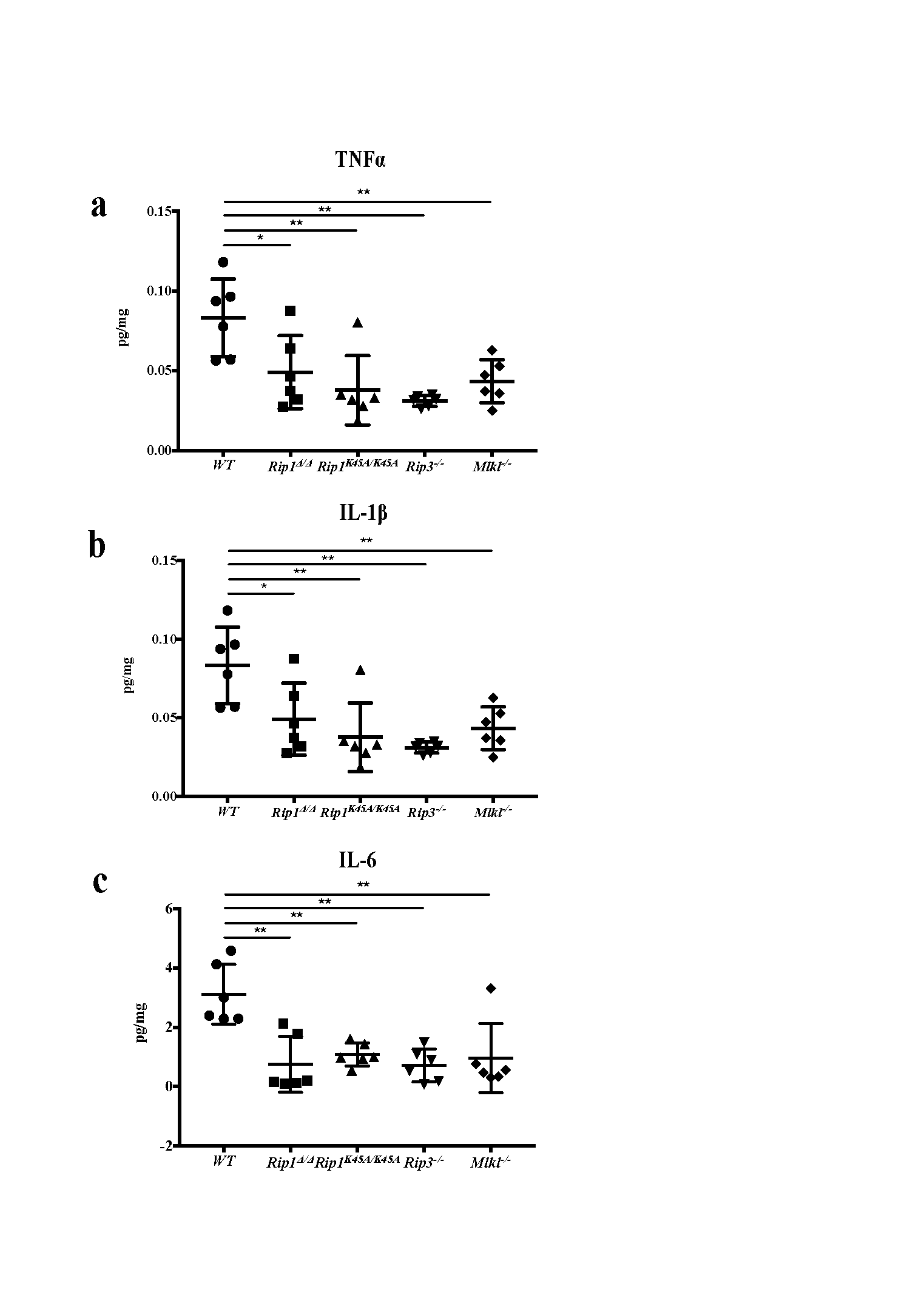

Supplement: Supplementary file 2 — Supplementary Figure 2 [file 41419_2020_2770_MOESM2_ESM.png]
