# Supplementary material for: Catalytically inactive RIP1 and RIP3 deficiency protect against acute ischemic stroke by inhibiting necroptosis and neuroinflammation
Source: Cell Death Dis. 2020 Jul 23;11(7):565. doi: 10.1038/s41419-020-02770-w (PMC7378260; doi:10.1038/s41419-020-02770-w)
Supplement: Supplementary file 3 — Supplementary Figure legends [file 41419_2020_2770_MOESM3_ESM.docx]

**Supplementary Figure Legends**

**Supplemental Figure 1** Cytokines and chemokines expression in serum of WT mice in sham group and MCAO/R group.

**Supplemental Figure 2** The protein expression of inflammatory factors in infarct area of *WT* mice and necroptosis loss function mice. Protein expression of TNFα(a), IL-1β(b) and IL-6(c) in infarct area of *WT* mice and necroptosis loss function mice ( *Rip1^K45A/K45A^*, *Rip1^Δ/Δ^*, *Rip3^-/-^* and *Mlkl^-/-^* mice) were detected by ELISA.
